# A Two-Component regulatory system with opposite effects on glycopeptide antibiotic biosynthesis and resistance

**DOI:** 10.1038/s41598-020-63257-4

**Published:** 2020-04-10

**Authors:** Rosa Alduina, Arianna Tocchetti, Salvatore Costa, Clelia Ferraro, Patrizia Cancemi, Margherita Sosio, Stefano Donadio

**Affiliations:** 1 Department of Biological, Chemical and Pharmaceutical Sciences and Technologies, University of Palermo, Viale delle Scienze, 90128 Palermo, IT Italy; 2 Naicons Srl, Via Ortles 22/4, 20139 Milan, Italy

**Keywords:** Antimicrobials, Industrial microbiology, Microbial genetics

## Abstract

The glycopeptide A40926, produced by the actinomycete *Nonomuraea gerenzanensis*, is the precursor of dalbavancin, a second-generation glycopeptide antibiotic approved for clinical use in the USA and Europe in 2014 and 2015, respectively. The final product of the biosynthetic pathway is an *O-*acetylated form of A40926 (acA40926). Glycopeptide biosynthesis in *N. gerenzanensis* is dependent upon the *dbv* gene cluster that encodes, in addition to the two essential positive regulators Dbv3 and Dbv4, the putative members of a two-component signal transduction system, specifically the response regulator Dbv6 and the sensor kinase Dbv22. The aim of this work was to assign a role to these two genes. Our results demonstrate that deletion of *dbv22* leads to an increased antibiotic production with a concomitant reduction in glycopeptide resistance. Deletion of *dbv6* results in a similar phenotype, although the effects are not as strong as in the Δ*dbv22* mutant. Consistently, quantitative RT-PCR analysis showed that Dbv6 and Dbv22 negatively regulate the regulatory genes (*dbv3* and *dbv4*), as well as some *dbv* biosynthetic genes (*dbv23* and *dbv24*), whereas Dbv6 and Dbv22 positively regulate transcription of the single, cluster-associated resistance gene. Finally, we demonstrate that exogenously added acA40926 and its precursor A40926 can modulate transcription of *dbv* genes but with an opposite extent: A40926 strongly stimulates transcription of the Dbv6/Dbv22 target genes while acA40926 has a neutral or negative effect on transcription of those genes. We propose a model in which glycopeptide biosynthesis in *N. gerenzanensis* is modulated through a positive feedback by the biosynthetic precursor A40926 and a negative feedback by the final product acA40926. In addition to previously reported control systems, this sophisticated control loop might help the producing strain cope with the toxicity of its own product. This work, besides leading to improved glycopeptide producing strains, enlarges our knowledge on the regulation of glycopeptide biosynthesis in actinomycetes, setting *N. gerenzanensis* and its two-component system Dbv6-Dbv22 apart from other glycopeptide producers.

## Introduction

**Figure 1:**
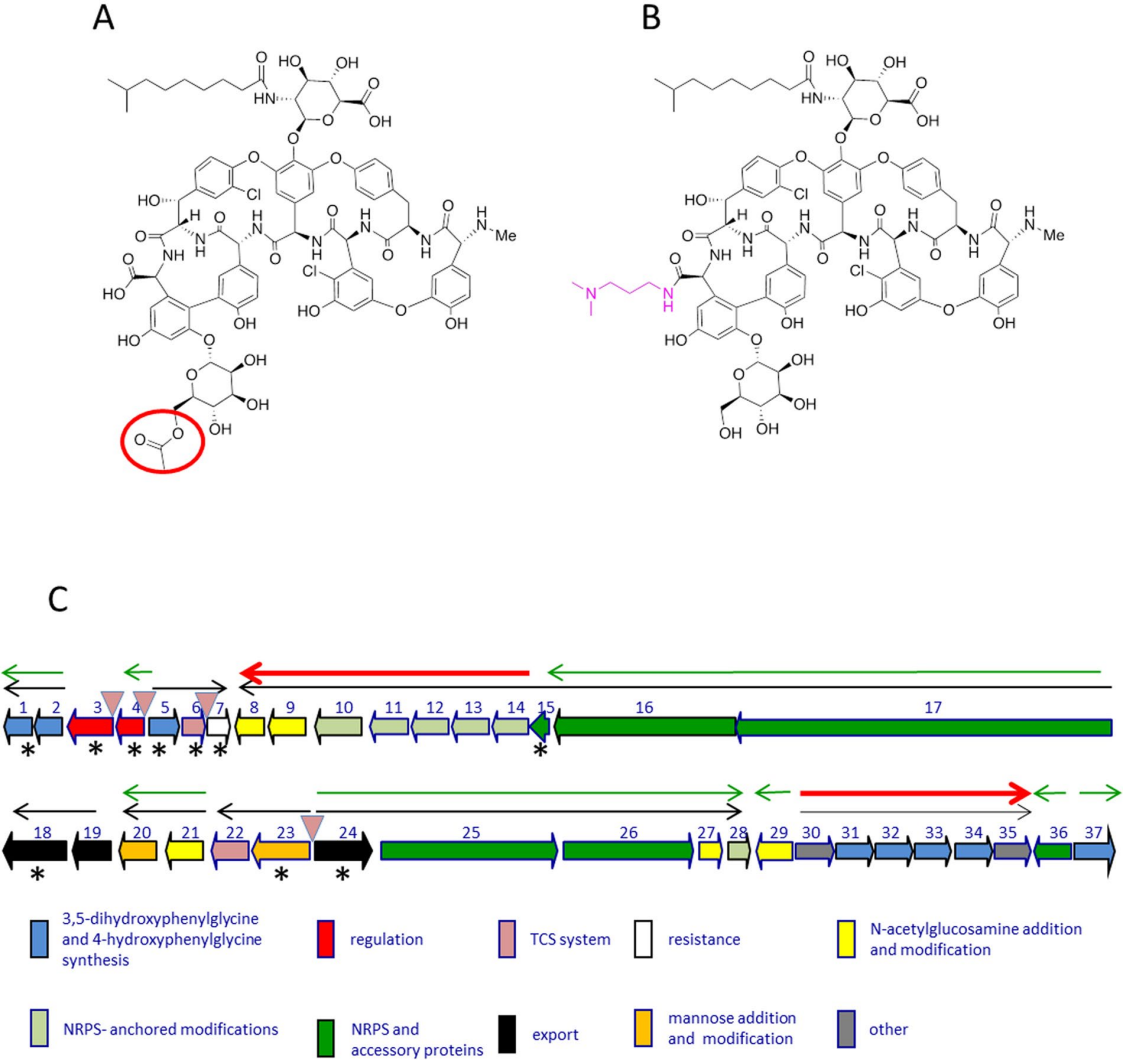
Chemical structures of O-acetyl A40926 (**A**) and of dalbavancin (**B**) and genetic organization of the *dbv* cluster (**C**). (**A,B**). Only the component B0 (the major congener in the A40926 complex) is shown for simplicity. The acetyl group of A40926 and the chemical modification present in dalbavancin are indicated in red circle and pink, respectively. (**C**) The thin black arrows indicate experimentally determined operons. Red and green arrows represent the transcriptional units controlled by Dbv4 and Dbv3, respectively. The *dbv* genes are grouped by functional category as indicated. Asterisks indicate the genes analyzed by qRT-PCR analysis in the present work.

The glycopeptide antibiotic A40926 (Fig. 1A) is the precursor for dalbavancin (Fig. 1B), a semisynthetic lipoglycopeptide clinically used for treatment of acute skin infections caused by methicillin-susceptible and methicillin-resistant *Staphylococcus aureus* and *Streptococcus pyogenes*.

A40926 is produced by the actinomycete *N. gerenzanensis* as a complex of related compounds, differing mostly by the type of *N*-acyl chain attached to the glucuronic acid moiety. The core structure of A40926 consists of a heptapeptide containing the proteinogenic amino acid tyrosine and the non-proteinogenic amino acids 3,5-dihydroxyphenylglycine (DPG) and 4-hydroxyphenylglycine (HPG). The heptapeptide is assembled by a nonribosomal peptide synthetase (NRPS) and modified by the action of various enzymes: four oxygenases, one halogenase, one hydroxylase, two glycosyltransferases, one hexose oxidase, one methyltransferase, one *N-*deacetylase, one *N-*acyltransferase and one *O*-acetyltransferase^1^. The form with the acetylated mannose (acA40926) is considered the final biosynthetic product^2^. However, mild extraction conditions are necessary to isolate acA40926. While A40926 and acA40926 are equipotent in antibacterial activity against most strains, the former is slightly more active against *S. aureus* and *Neisseria gonorrhoeae*^3^. Hence, the desacetylated form has been used as the substrate for dalbavancin semi-synthesis. In addition, as many methods for complex analysis involve alkaline treatment of the culture, which results in hydrolysis of the ester bond, past literature considered A40926 as the main fermentation product. Detailed studies of the regulation of glycopeptide biosynthesis in *N. gerenzanensis* will be crucial for the development of rational approaches towards overproduction of the dalbavancin precursor. The *dbv* gene cluster for acA40926 biosynthesis contains 37 protein-coding sequences (Fig. 1C) that participate in antibiotic biosynthesis, regulation, immunity, and export^1,4^. Specifically, the cluster encodes the positive regulators Dbv3 and Dbv4 that indirectly or directly activate different operons leading to acA40926 biosynthesis^5,6^. Dbv3 and Dbv4 belong to the LAL (large ATP binding regulators of LuxR) and the StrR family of regulators, respectively. The cluster also contains the putative response regulator *dbv6* and the sensor-kinase *dbv22*, whose products may be part of a two-component system (TCS), even if the genes are not organized in a bicistronic operon^4,7^. A relevant characterized TCS in actinomycetes is the *Streptomyces coelicolor* VanR (VanR_sc_) and VanS (VanS_sc_) system associated with a glycopeptide resistance cassette in this species, which however does not produce any glycopeptide.

In *S. coelicolor*, following vancomycin induction, the VanRSsc TCS activates transcription of the *vanHAX* genes, leading to the synthesis of peptidoglycan precursors terminating with D-alanyl–D-lactate (DAla–D-Lac),  causing a 1000-fold decreased binding affinity of vancomycin to its target^8–11^. Thus, in its induction mechanism and in the resulting peptidoglycan modification, the mechanism leading to glycopeptide resistance in *S. coelicolor* resembles that used by pathogenic *Enterococcus* spp^12,13^. While some glycopeptide producers do contain a *vanHAX* cassette, either associated with the gene cluster^14–16^ or elsewhere in the genome^17^, the *N. gerenzanensis* genome does not contain any such genes^18^. *N. gerenzanensis* must thus use different strategy(ies) to cope with the self-toxicity of its own product. Marcone and coworkers showed that the wild type strain synthesizes a peptidoglycan with 3–3 cross-linked peptide stems using the carboxypeptidase Dbv7^19^. Using substrates mimicking peptidoglycan, it was shown that Dbv7 cuts the D-Ala-D-Ala end of the peptidoglycan precursor on the outer surface of the cell membrane, mainly before and during antibiotic production^19,20^. However, deletion of *dbv7* in the original host or its heterologous expression result in measurable but modest differences in glycopeptide resistance^19^.

In this study, we characterized the function of *dbv6* and *dbv22* with respect to glycopeptide biosynthesis and resistance in *N. gerenzanensis* and established that *dbv* biosynthesis genes are under negative control by this TCS, while the resistance gene *dbv7* is under positive control. In addition, we discovered that exogenously added A40926 and acA40926 differently affect transcription of selected *dbv* genes. Based on the current results and those reported previously^2^, a complex model of regulation can be depicted with the late pathway intermediate A40926 and the end product acA40926 having different roles in controlling antibiotic biosynthesis and resistance.

## Results

### Dbv6 and Dbv22 are transcriptional repressors of A40926 biosynthesis

**Figure 2:**
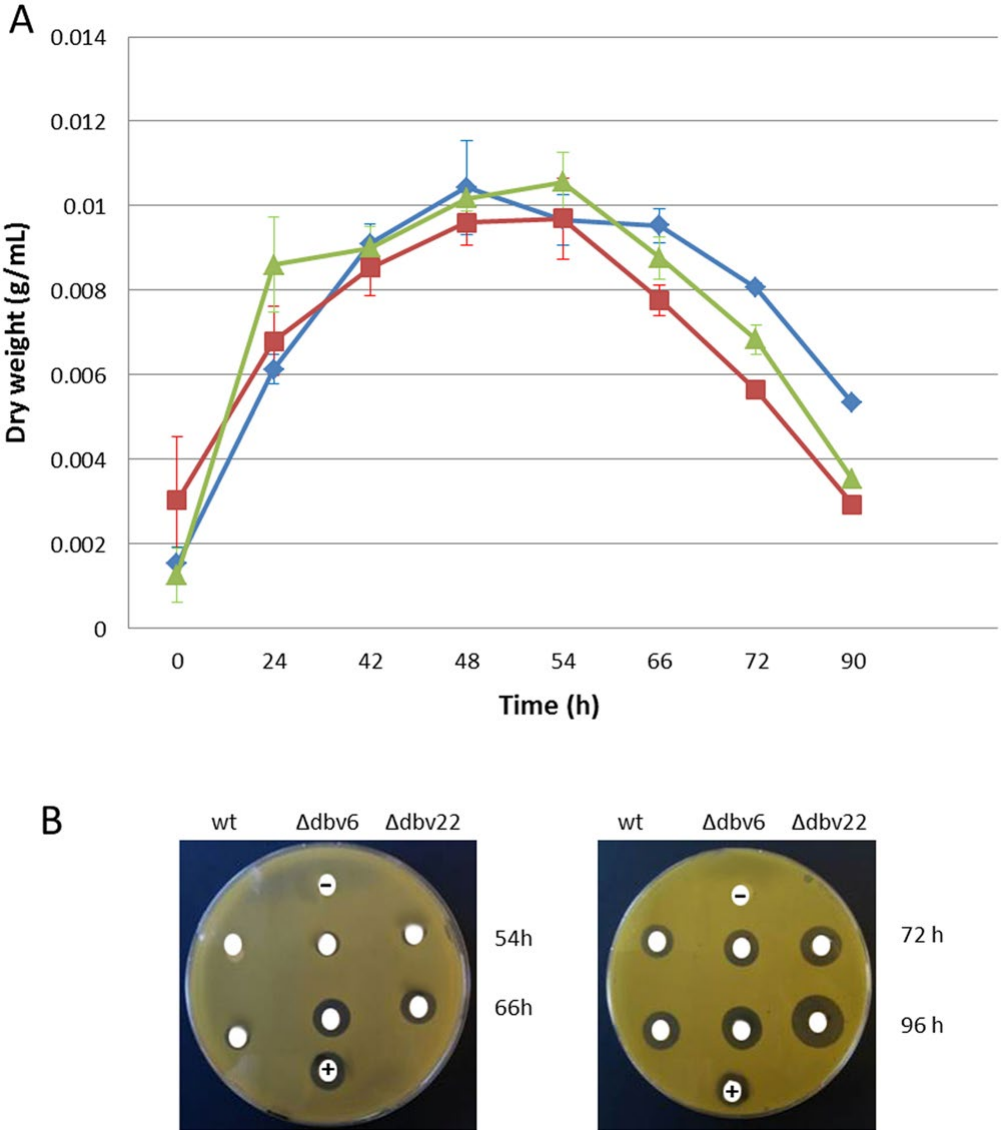
Growth curves and antibiotic production by *N. gerenzanensis* wild type, *Δdbv6* and *Δdbv22* strains in RARE3 medium. (**A**) The dry weight of the wild type, Δ*dbv*6 and Δ*dbv*22 strains are shown with green, red and blue lines, respectively. Standard deviation was calculated as average of three technical and two biological replicates. (**B**) Bioassays of 50 µL of the culture broth of the wild type, Δ*dbv*6 and Δ*dbv*22 strains using *K. rhizophila* as test strain. (+) and (−) indicate the positive (culture broth collected from the parental strain after 120 h) and negative (only growth medium) control, respectively.

*N. gerenzanensis* mutants defective in *dbv*6 and *dbv*22 were constructed as previously reported^6^ and as described under Material and Methods, respectively. When *N. gerenzanensis* was grown in a rich medium (RARE3) that affords good growth but moderate glycopeptide production, disruption of *dbv6* or *dbv22* did not significantly affect biomass accumulation (Fig. 2A). Interestingly, bioassays (Fig. 2B) of culture broth collected along the growth showed that antibiotic production started at 54 h in both mutants (12 h earlier than in the parental strain) and proceeded to higher levels up to 96 h, the last analyzed time point. Liquid chromatography–mass spectrometry (LC-MS) analysis of 96-h culture broths showed that the Δ*dbv6* and Δ*dbv22* mutants produced 52 and 70 µg/mL acA40926, respectively, in comparison with 34 µg/mL, as observed with the parental strain.

**Figure 3:**
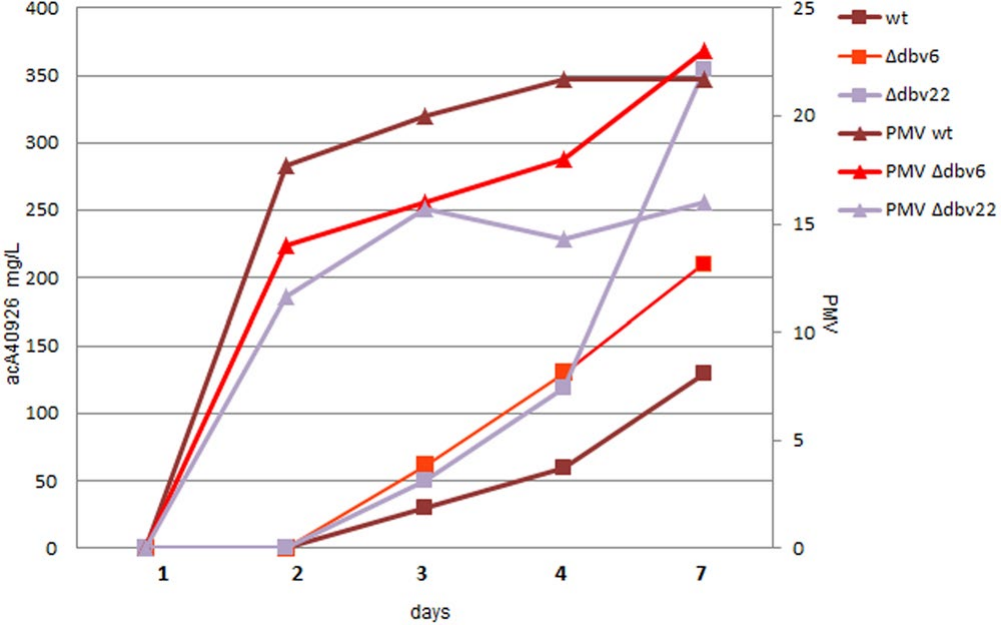
AcA40926 production and growth in V40P medium of the wild type, Δ*dbv6* and *Δdbv22* strains. Squares indicate acA40926 concentrations, while triangles represent %PMV. Brown, red and violet symbols correspond to the wild type, Δ*dbv6* and Δ*dbv22* strains, respectively.

When *N. gerenzanensis* was grown in a production medium (V40P), the higher production levels of the mutants were confirmed, with the Δ*dbv6* and Δ*dbv22* mutants producing 210 and 354 µg/mL acA40926 after 7 days, respectively, in comparison with 130 µg/mL seen in the parental strain (Fig. 3). Interestingly, under these conditions, we observed a small but consistent decrease in biomass accumulation, which was inversely related to the amount of produced glycopeptide. There was no difference in complex composition between the mutants and the wild type (Supplementary Fig. S1), with acA40926 as the main product at all time points in the three strains.

These results demonstrate that Dbv6 and Dbv22 negatively regulate glycopeptide production and their abolition leads to increased antibiotic production, despite a small decrease in biomass, indicating a substantial higher productivity of the mutants (in terms of µg of product per unit of biomass) in comparison to the parental strain. Across two different media, deletion of *dbv22* has a more prominent effect than *dbv6*.

**Figure 4:**
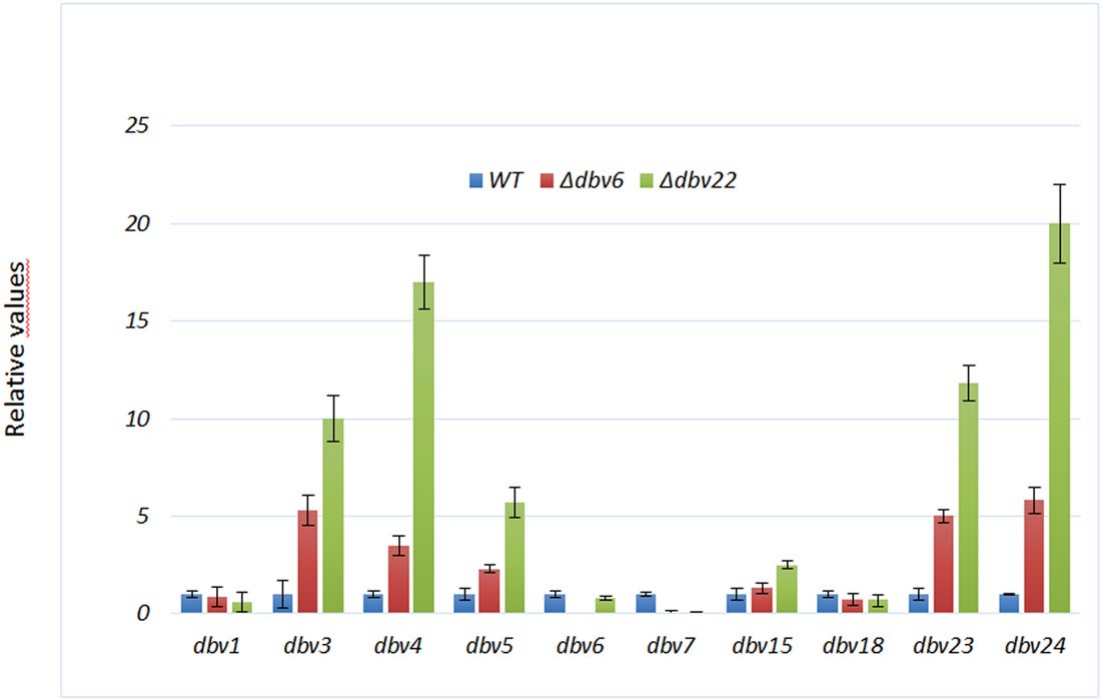
qRT-PCR of *dbv* genes in the wild type and *Δdbv6* and *Δdbv22* mutant strains. For each gene, data are normalized to the parental strain (blue bars). Red and green bars represent relative RNA levels seen in the Δ*dbv6* and Δ*dbv22* strains, respectively. Error bars are calculated from three independent determinations of mRNA abundance in each sample. RNAs were extracted after 54 h of growth in RARE3 medium.

Quantitative RT-PCR (qRT-PCR) analysis after 54 h of growth in RARE3 medium of the wild type and the two mutants was carried out to identify the Dbv6/Dbv22 target genes within the *dbv* cluster (Fig. 4). We focused our attention on ten genes as representatives of all the *dbv* operons. Besides the regulators *dbv3, dbv4* and *dbv6*, the analysis was carried out on the biosynthetic genes *dbv1* and *dbv5* (HPG synthesis), *dbv*23 (mannose acetylation) and *dbv15* (MbtH-like protein assisting NRPS action), on the ABC-transporter genes *dbv18*, and *dbv*24, and on the resistance gene *dbv7*^5,21^ (Fig. 1C). Consistent with the results from bioassays and LC-MS analysis, qRT-PCR analysis showed that transcription of some analyzed genes was strongly influenced by deletion of *dbv6* and more so by *dbv22* genes (Fig. 4). Indeed, the transcriptional levels of *dbv3, dbv4, dbv5, dbv23* and *dbv24* increased approximately 2–5 fold in the *Δdbv6* mutant and 5–20 fold in the Δ*dbv22* mutant; in contrast, *dbv7* was apparently not transcribed in both mutants. The transcriptional levels of *dbv1, dbv6*, and *dbv18* were not influenced by either mutation (Fig. 4).

### Dbv22 enhances glycopeptide resistance

**Figure 5:**
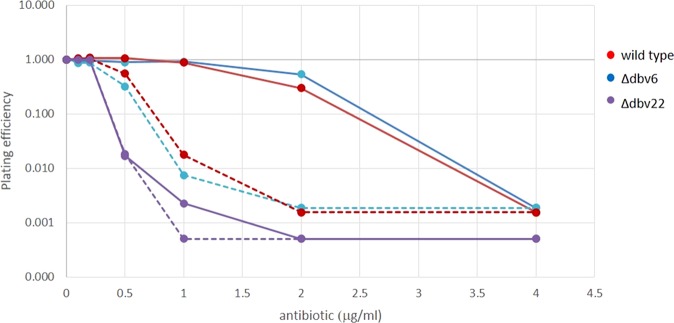
Resistance assay of the wild type and *Δdbv6* and *Δdbv22* mutant strains. Plating efficiency of the wild type, Δ*dbv6* and Δ*dbv22* strains on different concentrations of A40926 (solid lines) or of acA40926 (dashed lines). Reported data are the average of two independent counts. The limit of detection were 10^5^ CFU/mL for the wild type, Δ*dbv6*, and Δ*dbv22* strains.

We tested the wild type and the two mutant strains for sensitivity to A40926 and acA40926. Consistent with previous results^19^, the wild type strain formed no visible colonies when plated on medium containing 4 µg/mL A40926. Remarkably, it was more sensitive to acA40926 than to its desacetylated precursor, with a significant reduction in the ability to form colonies observed already at 0.5–1 µg/mL (Fig. 5). No significant differences between the wild type and the Δ*dbv6* mutant were observed. In contrast, the *Δdbv22* mutant strain was more sensitive to acA40926 and A40926, and it was able to form visible colonies only with up to 0.5 and 0.2 µg/mL of A40926 and acA40926, respectively (Fig. 5). Thus, consistent with qRT-PCR analysis, Dbv22 positively controls resistance to glycopeptides, likely by regulating expression of the carboxypeptidase Dbv7. The lack of an effect on glycopeptide resistance by the *dbv6* deletion might be due to the narrow range of glycopeptide concentration in which an effect is seen.

### A40926 strongly induces expression of *dbv* genes

**Figure 6:**
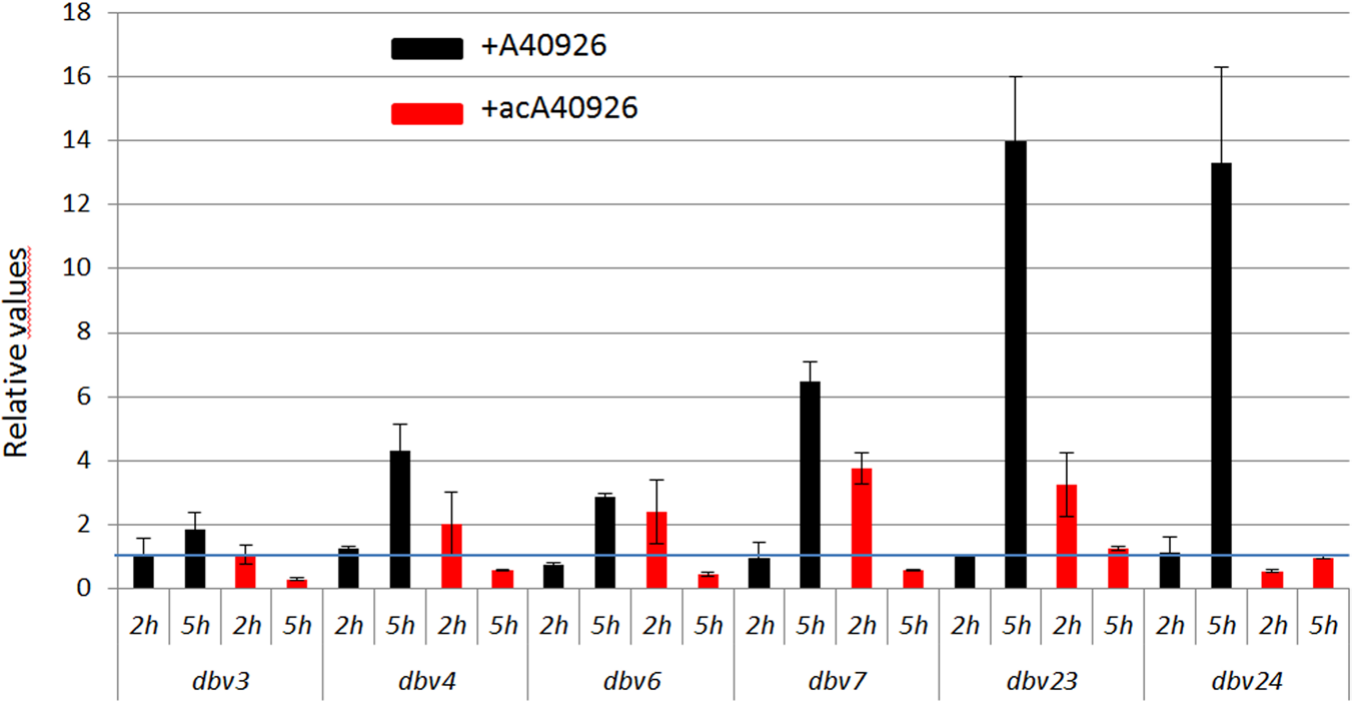
qRT-PCR analysis of the gene expression of *dbv3, dbv4, dbv6*, *dbv7, dbv23* and *dbv24* in the wild type strain. RNA was isolated from the wild type strain incubated in V40P medium for 2 and 5 h in the presence of 0.5 µg/mL A40926 (black bars) or 0.5 µg/mL acA40926 (red bars). Data are expressed as relative values to the no-addition controls. Within each sample, mRNA levels were normalized to *hrdB*, arbitrarily setting the ratio for each sample to 1. Standard deviations are calculated from three independent determinations of mRNA abundance in each sample.

The above results indicate that *N. gerenzanensis* can discriminate, in terms of sensitivity, between the highly related glycopeptides A40926 and acA40926. Previous work had also indicated that exogenously added acA40926, but not its desacetylated form, had an inhibitory effect on glycopeptide production^2^. We thus wondered whether exogenously added glycopeptides would have any effect on the transcriptional levels of the Dbv6/Dbv22 target genes. To this end, a 24 h culture of the parental strain inV40P medium was equally divided into three flasks, with each flask receiving either 0.5 µg/mL A40926, 0.5 µg/mL acA40926 or no addition. After further 2- and 5-h incubation, RNA was extracted and the transcriptional levels of all the regulatory genes (*dbv3, dbv4* and *dbv6*) and of the Dbv6-targeted genes (*dbv7*, *dbv23* and *dbv24*) were analyzed by qRT-PCR (Fig. 6). Addition of acA40926 had a modest inducing effect on the expression of the regulatory genes *dbv4* and *dbv6*, the resistance gene *dbv7* and the acetyltransferase gene *dbv23*, but only 2 h after compound addition. After 5 h, genes were slightly repressed (*dbv3*, *dbv4*, *dbv6* and *dbv7*) or unaffected (*dbv23* and *dbv24*). In contrast, addition of A40926 did not significantly affect transcription levels after 2 h. However, after 5 h all the analyzed genes were induced, with transcription levels 2-fold (for *dbv3*), 3–6-fold (for *dbv4*, *dbv6* and *dbv7*) or 13–14-fold (for *dbv23* and *dbv24*) higher relative to uninduced cells. qRT-PCR of the analyzed *dbv* genes in all three cultures before induction indicated modest flask-to-flask variation (Supplementary Fig. S2).

The above experiments were performed using the wild type strain, which does produce acA40926 and related pathway intermediates, with exogenous compounds added during early exponential growth, when glycopeptide levels are below the limit of detection (about 1 µg/mL). When a similar experiment was carried out with the Δ*dbv4* mutant strain, which does not produce any glycopeptide^6^, we could not detect any transcription of *dbv23* and *dbv24*, suggesting that these genes might be indirect targets of Dbv4 (Supplementary Fig. S3). Thus, one or more components of the glycopeptide complex or intermediates thereof could exert a positive feedback on the transcription of at least some *dbv* genes.

### Direct control of Dbv6 on *dbv4* and *dbv7* gene expression

**Figure 7:**
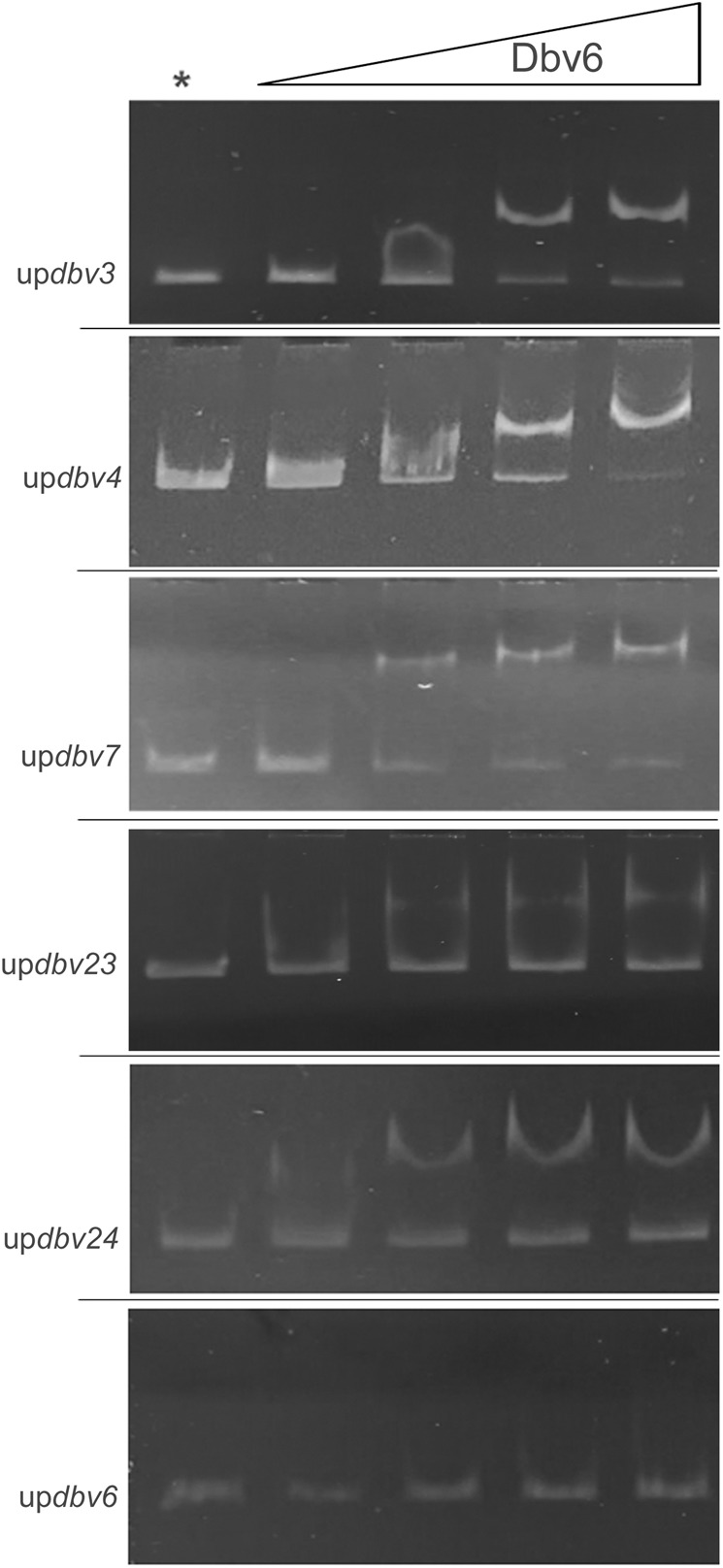
Gel mobility shift assays of DNA regions upstream of *dbv*3, *dbv*4, *dbv7*, *dbv23, dbv24* and *dbv6*. Lanes labeled with an asterisk contained the probe only (up*dbv3*, up*dbv4*, up*dbv7*, up*dbv23*, up*dbv24* and up*dbv6*). All lanes contained 50 ng of target DNA. Increasing concentrations of His-Dbv6 (0.5, 1, 1.5, 2 mM) were incubated in the presence of probes as indicated.

We investigated whether Dbv6 can bind to the upstream region of the target genes and directly control their expression. Gel mobility shift assays of the regions upstream to *dbv3, dbv4, dbv6, dbv7, dbv23* and *dbv24* were carried out using a purified His-tagged Dbv6 protein overexpressed in *E. coli*. These assays did reveal a binding activity of Dbv6 towards the upstream regions of the regulatory genes *dbv3* and *dbv4*, of the resistance gene *dbv7*, of the genes *dbv23-dbv22*, coding for the acetyltransferase and the sensor kinase, and of the operon *dbv24-dbv28*, which encodes an ABC-transporter, two NRPS modules, the *N-*methyltransferase and the enzyme involved in β-hydroxylation of the tyrosine residue (Fig. 7). No shift was detected using the upstream region of *dbv6* (Fig. 7). Control experiments using the internal region of the vegetative sigma factor encoding gene (Supplementary Fig. S4) further confirmed the specificity of DNA binding by Dbv6 observed on some of the target genes. Thus, these data are consistent with Dbv6 directly controlling expression of the target genes. Further studies will be necessary to understand how Dbv6 exerts positive and negative control on different DNA regions.

### Dbv6 and Dbv22 are unrelated to other TCSs associated with other glycopeptide gene clusters

**Figure 8:**
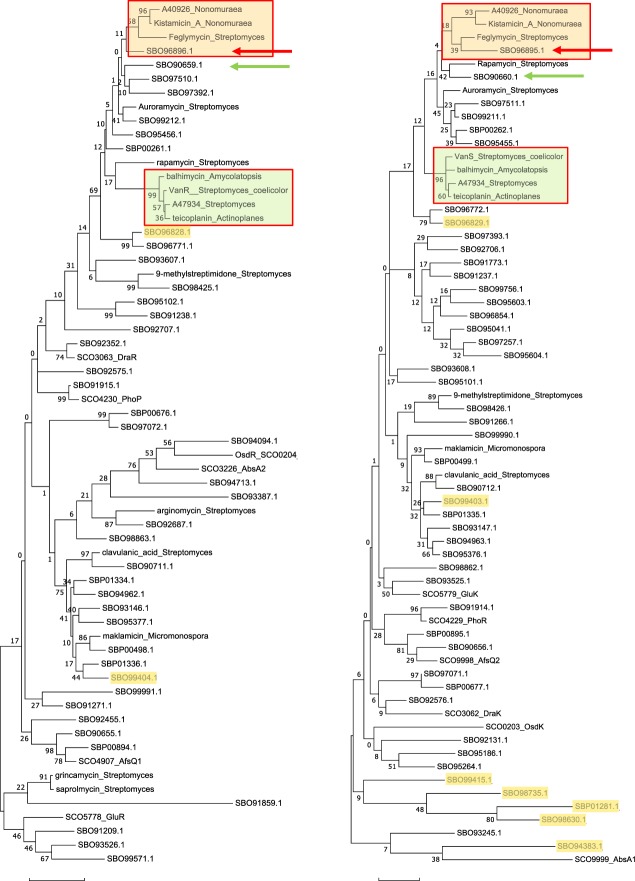
Phylogenetic analysis of 62 response regulators (left panel) and 65 sensory kinases (right panel). Sequences associated with established gene clusters are identified by the compound name followed by the genus of the corresponding strain; characterized *S. coelicolor* proteins are identified by the protein name followed by the SCO code; and *N. gerenzanensis* sequences are denoted by the SBO or SBP codes. The branches associated with the Van-and Dbv-related TCSs are identified by green and orange boxes, respectively. Green and red arrows indicate the adjacent members of two different, uncharacterized *N. gerenzanensis* TCSs closely associated with the Dbv branches. Sequences associated with *N. gerenzanensis* biosynthetic gene clusters, as identified by AntiSMASH 5.0^42^, have a yellow highlight.

TCSs are often associated with glycopeptide gene clusters, where they have been shown in some cases to play a role in glycopeptide resistance. They appear similar to the well-characterized *S. coelicolor* VanRS system, which plays a role in glycopeptide resistance in a non-producing strain^9^. However, reciprocal Blast searches indicate that Dbv6 and Dbv22 are not orthologs of VanR_sc_ and VanS_sc_, respectively. Inspired by recent work on phylogenetic analysis of cluster-associated StrR- and LuxR-related regulators^22^, we performed a phylogenetic analysis of TCSs from actinomycetes. To this end, we retrieved response regulators and sensory kinases associated with characterized actinobacterial gene clusters from the MiBIG database^23^, well-characterized TCSs from *Streptomyces coelicolor*^24,25^ and the response regulators and sensory kinases identified in the *N. gerenzanensis* genome^18^ (Fig. 8).

With respect to the response regulators, those associated with the balhimycin, teicoplanin and A47934 clusters branch together along with *S. coelicolor* VanR (Fig. 7, left panel). In contrast, Dbv6 clusters together with response regulators associated with gene clusters for kistamicin A (from a *Nonomuraea* sp^26^) and for feglymycin (from a *Streptomyces* sp^27^.). Kistamicin A is a partially cross-linked heptapeptide sharing some amino acid residues with glycopeptide antibiotics, whereas feglymycin is a linear tridecapeptide containing several HPG and DPG residues. Both compounds have antiviral activity but modest antibacterial activity in comparison with glycopeptide antibiotics^28–30^.

A similar picture emerges when analyzing the sensory kinases: those associated with the balhimycin, teicoplanin and A47934 clusters, and *S. coelicolor* VanS form a separate branch from the one containing Dbv22 and the kistamicin A- and flegymycin-associated sequences (Fig. 8, right panel). Interestingly, the adjacent members of two different, uncharacterized TCSs encoded by the *N. gerenzanensis* genome cluster closely with the Dbv6 and Dbv22 branches, suggesting these proteins might respond to similar stimuli and/or be regulated by similar factors. It should be noted that all the other sensory kinases or response regulators from *N. gerenzanensis* associated to a putative biosynthetic gene cluster are unrelated to Dbv6 or Dbv22.

## Discussion

The results presented here provide evidence that the response regulator Dbv6 and the sensor kinase Dbv22 negatively control A40926 production and that Dbv22 positively controls A40926 resistance. These effects are associated with altered transcription levels. Indeed, strains deleted for these genes are upregulated in the expression of the two key regulators *dbv3* and *dbv4*. Conversely, the *Δdbv22* mutant strain expresses less *dbv7*, the only resistance gene present in the *dbv* cluster. This would suggest that Dbv6 and Dbv22 represent the two components of a signal transduction system responding to some stimuli and controlling key elements of the *dbv* cluster. Remarkably, deletion of *dbv22* can lead to the paradoxical result of a mutant producing increased glycopeptide levels while becoming more sensitive to its own product.

While TCSs are frequently found in glycopeptide gene clusters, different regulatory strategies are apparently adopted by glycopeptide-producing actinomycetes. Abolition of the response regulator or of the sensor kinase gene in *Streptomyces toyocaensis* led to increased sensitivity to A47934^11^. In *Amycolatopsis balhimycina*, the deletion of genes for the VanRS-like TCS VnlRSAb affected VanY expression in a Δ*van*HAX background^31^ while did not influence balhimycin production^17,31^. In the glycopeptide non-producing *S. coelicolor*, deletion of *vanR* but not of *vanS* led to a strain sensitive to vancomycin^11^. Apart from the VanR genes associated with a *vanHAX* resistance cassette (e.g, those from *S. coelicolor*, *S. toyocaensis* and *Actinoplanes teichomyceticus*), the gene(s) targeted by VanR in *A. balhimycina* remain to be identified. Here, we demonstrate that in *N. gerenzanensis* direct target genes of Dbv6 are the regulatory genes *dbv3* and *dbv4*, and the genes *dbv23-dbv22*, coding for the acetyltransferase and the sensor kinase, and the *dbv24-dbv28* operon, since the transcription of all these genes is induced in the *Δdbv6* mutant background and that Dbv6 binds to the upstream regions of these genes. Another direct target of Dbv6 but regulated in the opposite manner is the peptidase *dbv7* (Fig. 7).

The impacts of the *dbv6* and *dbv22* mutations are quantitatively different. First of all, the relative expression levels of the analyzed *dbv* genes (encoding the positive regulators Dbv3 and Dbv4, the prephenate dehydrogenase Dbv5, the acetyltransferase Dbv23 and the ABC-transported Dbv24) in the *Δdbv6* mutant are somehow intermediate between those of the parental strain and of the *Δdbv22* mutant (Fig. 4). In terms of antibiotic resistance, the *Δdbv22* strain is marginally but consistently more sensitive than the parental strain and the *Δdbv6* mutant to A40926 and acA40926. Finally, when *N. gerenzanensis* is grown in production medium, the *Δdbv6* and the *Δdbv22* mutants show, up to 48 h, an identical but slightly higher acA40926 production rate than the parental strain, with *Δdbv22* keeping an equivalent rate up to 7 days and reaching acA40926 levels (350 µg/mL) significantly higher than *Δdbv6* and the parental strain (200 and 130 µg/mL, respectively; Fig. 3). Remarkably, higher production levels by *Δdbv22* are achieved despite a slightly lower biomass accumulation (Fig. 3).

The fact that all the analyzed parameters are more strongly altered in the *Δdbv22* than in the *Δdbv6* background suggests that Dbv22 and/or Dbv6 might interact with other response regulators and/or sensor kinases, respectively, present in the cell. Indeed, the *N. gerenzanensis* genome harbors 43 and 47 TCS pairs, some of them highly related to VanR and VanS (Fig. 8). A homologous of *dbv6* gene is present in the *N. gerenzanensis* genome. The gene is located about 4.5 Mbp away from Dbv6, with which it shows 68% sequence identity and 77% similarity. All 8 diagnostic residues of the phosphorylation site are invariant with Dbv6. The HTH domain is highly conserved.

Marcone and coworkers^19^ have previously shown that *N. gerenzanensis* is essentially sensitive to A40926, that the carboxypeptidase VanY (i.e., Dbv7) provides some protection but that insensitivity to glycopeptides is mostly acquired during growth by some unknown mechanism. That some other resistance mechanism operates in *N. gerenzanensis* is consistent with our observed lack of transcription of *dbv7* in the *Δdbv6* strain (Fig. 4), a mutant that is as glycopeptide resistant as the parental strain (Fig. 5). Thus, *dbv6* does not apparently contribute to glycopeptide resistance while *dbv22* does, suggesting that a second resistance mechanism influenced by Dbv22 takes over. The complex interplay of glycopeptide production and resistance is also highlighted by the fact that deletion of *dbv7* did not affect A40926 production, as reported by Marcone and coworkers^19^.

When added just at 24 h of growth, when no glycopeptide production is detected yet, 0.5 µg/mL A40926 can strongly induce expression of all tested *dbv* genes, particularly *dbv23* and *dbv24*. AcA40926 has the opposite effect. The simplest explanation of this observation is that A40926, but not acA40926, binds to a receptor to activate signal transduction, relieving inhibition or leading to a massive transcription of *dbv* genes. It is tempting to speculate that the receptor that A40926 binds to is actually Dbv22. Indeed, the ~14-fold stimulation of *dbv24* upon A40926 addition is remarkably similar to its upregulated expression in the Δ*dbv22* background. Sensor kinases have been shown to be able to recognize and respond to glycopeptides: VanS_sc_ is induced by vancomycin but not by teicoplanin and A47934^8,11^, while the *S. toyocaensis* VanS sensor kinase is induced by A47934 but not by vancomycin and teicoplanin^11^.

In earlier work, we showed that a strain defective in mannose acetylation produces increased levels of A40926 in comparison to the wild type and that this enhanced production can be reverted by adding acA40926 – but not A40926 – to the culture at time zero, while addition of acA40926 had no effect on the wild type^2^. The present and previous results are consistent assuming that acA40926 can also bind to the same receptor but it cannot trigger its activation. Thus, acA40926 would compete with A40926 for the same binding site, acting as an apparent inhibitor of glycopeptide production. Discrimination between the acetylated and desacetylated form of the glycopeptide by *N. gerenzanensis* can also be observed in the resistance profile of the wild type, which is more sensitive to acA40926 than A40926 (Fig. 5).

The data presented here and in previous work^2,5,6^ allow us to refine a possible model for regulation of glycopeptide production in *N. gerenzanensis*^1^. When cells are actively growing, transcription of *dbv3* and *dbv4* is repressed by the direct action of Dbv6, as well as by other factors related to growth (nutritional factors, or quorum sensing systems could control A40926 biosynthesis)^5^. At the same time, the resistance gene *dbv7* is expressed as a safeguard mechanism against small amounts of glycopeptides that might be produced. As growth slows down, expression of the master regulators Dbv3 and Dbv4 increases so that some glycopeptide synthesis can occur. When sufficient A40926 accumulates, repression mediated by Dbv6/Dbv22 is relieved and further expression of the *dbv* genes can occur, including that of the acetyltransferase *dbv23* and of the ABC transporter *dbv24*, which results in formation of acA40926 form and its export outside of the cell, where it can compete out A40926 for Dbv22 binding, eventually restoring repression exerted by Dbv6/Dbv22.

This mechanism is somehow reminiscent of the feed forward mechanism proposed for microbisporicin/NAI-107 production in *Microbispora corallina*, where a late pathway intermediate can activate production^32^ through the activation of the ECF sigma factor σMibX, sequestered at the membrane level by an antisigma factor. An unknown signal should lead to production of microbisporicin, the inactivation of the anti-sigma factor, the release of σ^MibX^, and high-level expression of the entire gene cluster. Interestingly, both A40926 and NAI-107 target lipid II^33^, are produced by two strains belonging to different genera of the order *Streptosporangiales* that contain 3–3 cross-linked dimers in their cell wall and that are highly sensitive to their own product^33^.

## Material and methods

### Bacterial strains and culture conditions

The bacterial strains and plasmids used in this study are listed in Table 1. *Escherichia coli* strains were grown in Luria broth liquid medium at 37 °C and supplemented with 50 µg/mL apramycin when necessary to maintain plasmids.

**Table 1:** Bacterial strains, plasmids and cosmids used in this study.

Strain or plasmid	Description	Source or reference
**Bacterial strains**		
*Nonomuraea gerenzanensis*	Wild type	ATCC 39727
*Δdbv6* mutant	*N. gerenzanensis* strain in which the *dbv6* gene was replaced with an apramycin cassette	^6^
*Δdbv22* mutant	*N. gerenzanensis* strain in which the *dbv22* gene was replaced with an apramycin cassette	This study
*Kocuria rhizophila*	Used for bioassay of A40926	ATCC 9341
*Escherichia coli* DH10B	F_ *endA1 recA1 galE15 galK16 nupG rpsL* Δ*lacX74* Δ80*lacZ*ΔM15 *araD139* Δ(*ara-leu*)*7697 mcrA* Δ(*mrr-hsdRMS-mcrBC*) λ^-^; host for routine subcloning experiments	Invitrogen
*Escherichia coli* ET12567/pUZ8002	(*dam-13*::Tn*9 dcm-6*) pUZ8002^+^(Δ*oriT*); used for conjugative transfer of DNA and for demethylating plasmid DNA	^44^
*Escherichia coli* BW25113/pIJ790	Δ (*araD-araB*)567 Δ*lacZ4787* (::*rrnB-4*) *lacIp-4000* (*lacI*q) λ^-^ *rpoS369* (Am) *rph-1* Δ (*rhaD-rhaB*) *568 hsdR514*: used to generate recombinant cosmid 1B1	^45^
**Plasmid/cosmid**		
1B1	Cosmid containing part of the *dbv* cluster from *dbv*17 to *dbv*37	^4^
1B1Δ*dbv22*	Derivative of 1B1 with the inactivated *dbv*22 gene	This study
pIJ773	pUC19 containing the *aac(3)IV-oriT* cassette; source of the *aac(3)* gene	^37^

Cell stocks of the *N. gerenzanensis* strains were prepared from cultures in RARE3 medium as already described^34^ and stored at −80 °C. Liquid cultures were prepared by inoculating 0.2 mL of the frozen stock into 20 mL of RARE3 medium; after 120 h of growth, 1 mL of this culture was inoculated into 50 mL of fresh RARE3 medium in an orbital shaker (500 × g) in 250-mL baffled flasks at 30 °C. To detect glycopeptide production, samples of culture broths at different time points were withdrawn and stored at −80 °C before analysis. Cultivation in glycopeptide production medium was performed as already described^2^. Biomass was determined by measuring the cell dry weight of mycelial pellet recovered from a 1-mL culture sample after drying the pellet at 65 °C for about 24 h. Alternatively, the relative packed mycelial volume (%PMV) was determined after centrifugation of a 6-mL culture sample for 10 min at 4000 × g.

For assessing induction by glycopeptides, the parental strain or the *Δdbv4* mutant were cultivated in V40S medium as above and then transferred into (fresh) V40P medium^2^. After 24 h at 30 °C, the culture was equally divided into three flasks, and either 0.5 µg/mL A40926 or 0.5 µg/mL acA40926 were added, with the third flask used as no-addition control. After further 2 and 5 h, 1-mL samples were collected and centrifuged to recover cells. RNA was extracted and processed as below.

### Analysis of antibiotic production

Antibiotic production of the mutant strains was firstly evaluated using the paper disc diffusion method and *Kocuria rhizophila* as the assay organism as described previously^35^. For high-performance liquid chromatography (HPLC) and liquid chromatography-mass spectrometry (LC-MS) analyses, 500 µL aliquots of broth cultures were collected at the time points indicated and treated as reported previously^6,36^.

### Construction of the Δdbv22 strain

PCR targeting^37^ was applied to replace *dbv22* with a cassette containing an apramycin-selectable marker using pIJ773 as template and the PCR primers reported in Table 2. *E. coli BW25113/pIJ790* bearing cosmid 1B1^4^ was electrotransformed with the deletion cassette. Intergeneric conjugation was carried out as previously reported^6^. Exconjugants were analyzed by PCR (Supplementary Fig. S5) using primers reported in Table 2. One out of eleven clones analyzed contained the interrupted *dbv22* gene and used for further studies. Sequencing of the PCR product confirmed that the apramycin-resistant cassette was correctly inserted in the target gene through homologous double-cross recombination.

**Table 2:** Primers used in this study.

Gene(s)	Forward (5′-3′)	Reverse (5′-3′)	Predicted size (bp)
**Used in RT-PCR or qRT-PCR**			
*hrdb*	ctgatcaacaaggtcgcccg	gccgtacctctgcacctcg	129
*16S*	agcttgttggtggggtagtg	tcaccgctacaccaggaatt	470
*dbv3*	gaagaggttgcccaggtcac	ccgaggcgctctacatcac	101
*dbv4*	cagtaattccggcttggtcc	aaacaggtcggcatctccc	114
*dbv5*	gtggacctggcattgatcg	ctggtcacatcggtgtacgc	101
*dbv6*	agctgtgcgctgagatcg	acggcttcggcagatagtc	126
*dbv7*	ccagctcgtcggtctcac	cccttgacgtggttggac	141
*dbv23*	gaccgcccacttgttgatc	ggcagctcaccacctacg	110
*dbv24*	tcggaggtttgctgaagg	agatcgaatccaatgcgg	147
**Used to prepare probes for EMSA**			
*upstream 3*	gaatcgagcaacctcgtcag	gcaggaatgggaaaggatct	300
*upstream 4*	gcatcggtcttatccgaaac	cgtcgcatcctcttttcttc	226
*upstream 6*	tggtcgaggtctccgtgc	cattcgtgtcgccgtctc	150
*upstream 7*	tgaaggcgacgatcaacc	tcctcatccctctcctccag	188
*upstream 23*	gtccaggtgaacgcttggt	ccttcagcaaacctccgaac	178
*upstream 24*	gcaggtcggaagggacatc	gtgaccaagcgttcacctgg	176

### Resistance assay

The ability to form colonies of the *N. gerenzanensis* wild type, *Δdbv6* and *Δdbv22* strains was assessed by plating serial dilutions of stock cultures (prepared from frozen mycelium) on 0.25X BTT plates^38^ containing 0, 0.1, 0.2, 0.5, 1.0, 2.0 or 4.0 µg/mL A40926 or acA40926. Two replicate plates were used for each antibiotic concentration. After 6 days at 30 °C, the number of colony forming units (CFU) was recorded.

### Total RNA isolation and qRT-PCR analysis

To perform transcriptional analysis, a 6-mL sample was withdrawn from a 30-mL culture. The mycelium was harvested by centrifugation and RNA was extracted using the RNeasy midi kit (Qiagen), followed by treatment with DNase I (Roche) according to the manufacturer’s instructions. RNA quality was previously checked using Reverse transcription (RT)-PCR analysis as previously described^39^. It was converted to cDNA as already described^34^ and amplified using primers reported in Table 2. As control genes, *hrdB*, encoding a vegetative sigma factor, or 16S rRNA, were used. Gene expression was analyzed quantitatively by using the 7300 real-time PCR system (Applied Biosystems) with SYBR green PCR master mix (Applied Biosystems) in 96-well plates. Two microliters of cDNA was added to 20 µL of the PCR mixture. Amplification required activation of AmpErase UNG at 50 °C for 2 min, followed by denaturation at 95 °C for 10 min and then 40 cycles at 95 °C for 15 s and 60 °C for 1 min.

### Overexpression and purification of recombinant protein His-Dbv6

*Escherichia coli* DH10B and BL21 (Invitrogen) were used in this study. Plasmids pGEM-T (Promega) and pRSETB (Invitrogen) were used for cloning PCR products, and protein expression, respectively. *N. gerenzanensis* chromosomal DNA was used as template to amplify *dbv*6 by PCR using the primers 5’-aaaaggatccaatgcgcgttctggtggtgga-3’ and 5’-ttttaagctttcagatgcgatatccctcgc-3’ (underlines indicate the *BamHI* and *HindIII* sites, respectively). The amplified fragment was digested with *BamHI* plus *HindIII* and ligated into the *BamHI* and *HindIII* sites of pRSETB, yielding pRSET-Dbv6, which was introduced into BL21(DE3)pLysS cells by heat shock treatment^40^. Plasmid pRSET-Dbv6 expresses the entire Dbv6 protein with a His6 tag at its N terminus under the control of the T7 promoter and the *lac* operator. Fidelity of PCR amplification was confirmed by DNA sequencing. Dbv6 overexpression and purification were performed using the procedures already described for Dbv4^5^.

### Gel mobility shift assay

The gel mobility shift assay was performed according to the method already described^41^. Briefly, for the binding assay, increasing amount of Dbv6 (2.8 to 14 µg) was incubated for 10 min at 4 °C in 20 μL of 12.5 mM Tris-HCl (pH 7.5), 10% glycerol, 62.5 mM KCl, 0.75 mM DTT, and 5 mM MgCl2. After 15 min of incubation with 100 ng of DNA, complexes and free DNA were resolved on non-denaturing 5% polyacrylamide gels run in 0.5× Tris-borate-EDTA buffer at 150 V for approximately 2 h and then stained in a bath containing ethidium bromide. The DNA probes were amplified by PCR using the primers listed in Table 2. BSA and the internal region of *hrdB* were used in control experiment (Supplementary Fig. 4).

### Phylogenetic analysis

Thirteen response regulators and ten sensory kinases were retrieved from the MiBIG database of characterized actinobacterial biosynthetic gene clusters^23^; and forty-one response regulators and forty-seven sensory kinases were identified by blast searches of the *N. gerenzanensis* genome^18^. Seven characterized TCSs from *S. coelicolor* (AbsA, Afs, Dra, Glu, Osd, Pho and Van)^24,25^ were also included. Sequences associated with *N. gerenzanensis* biosynthetic gene clusters were identified by AntiSMASH 5.0^42^. Sequence alignments and phylogenetic analyses were performed with the MEGA 7 package^43^.

## Supplementary information

Supplementary Information.
