# Supplementary material for: A Two-Component regulatory system with opposite effects on glycopeptide antibiotic biosynthesis and resistance
Source: Sci Rep. 2020 Apr 10;10:6200. doi: 10.1038/s41598-020-63257-4 (PMC7148328; doi:10.1038/s41598-020-63257-4)
Supplement: Supplementary file 1 — Supplementary Information. [file 41598_2020_63257_MOESM1_ESM.pdf]

A Two-Component regulatory system with opposite effects on glycopeptide antibiotic biosynthesis and resistance

Rosa Alduina[1#], Arianna Tocchetti[2], Salvatore Costa[1], Clelia Ferraro[1], Patrizia Cancemi[1], Margherita Sosio[2], Stefano Donadio[2]

1) Department of Biological, Chemical and Pharmaceutical Sciences and Technologies, University of Palermo, Viale delle Scienze, 90128 Palermo, IT.

2) Naicons Srl, Via Ortles 22/24, 20138 Milan, Italy

[*]**Corresponding author**: Rosa Alduina, Department of Biological, Chemical and Pharmaceutical Sciences and Technologies (STEBICEF), Università degli Studi di Palermo, Viale delle Scienze Bd.16, 90128 Palermo, Italy. valeria.alduina@unipa.it. +3909123897306; Work fax number: 091 6577210

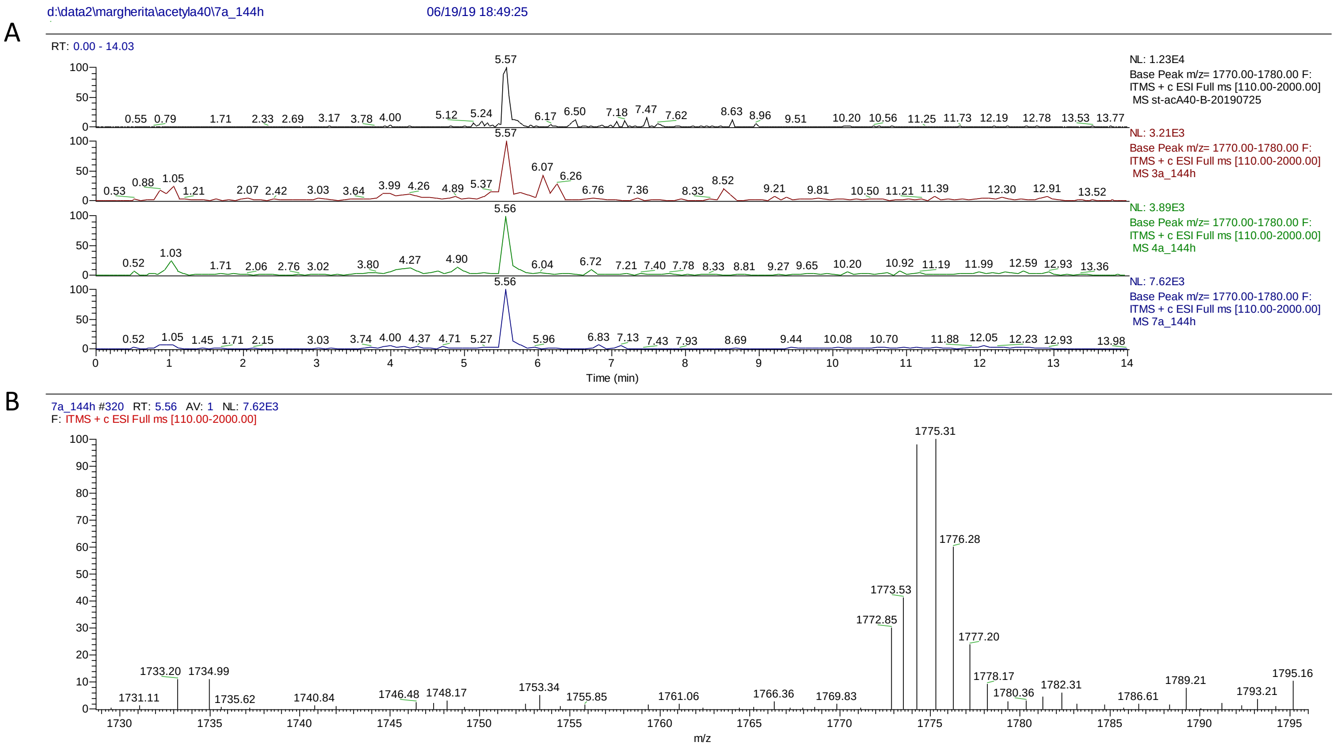

Supplementary Figure S1. Metabolite analysis of the wild type, Δ*dbv*6 and Δ*dbv*22 strains.

The extracted ion chromatograms of acA40926 (m/z 1775 [M+H]+) from standard (black line) and from cultures of WT (red line), of Δ*dbv*6 (green line) and of Δ*dbv*22 (blue line) strains are reported in Panel A. Mass spectra for acA40926 is reported in Panel B.

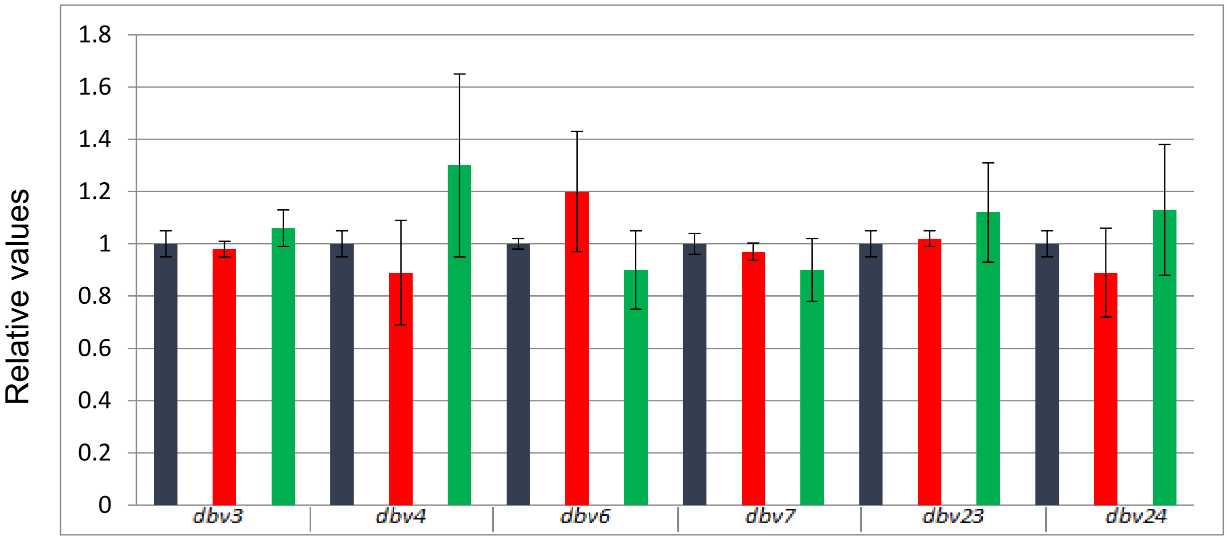

Supplementary Figure S2. qRT-PCR analysis of *dbv3, dbv4, dbv6*, *dbv7, dbv23* and *dbv24* in the cultures of Fig. 6 immediately before glycopeptide addition.

Blue, red and green bars represent cultures containing no-addition, 0.5 µg/mL A40926 and 0.5 µg/mL acA40926, respectively. mRNA levels are expressed as relative values to *hrdB*, arbitrarily setting the ratio for the no-addition sample to 1. Standard deviations are calculated from three independent determinations of mRNA abundance in each sample.

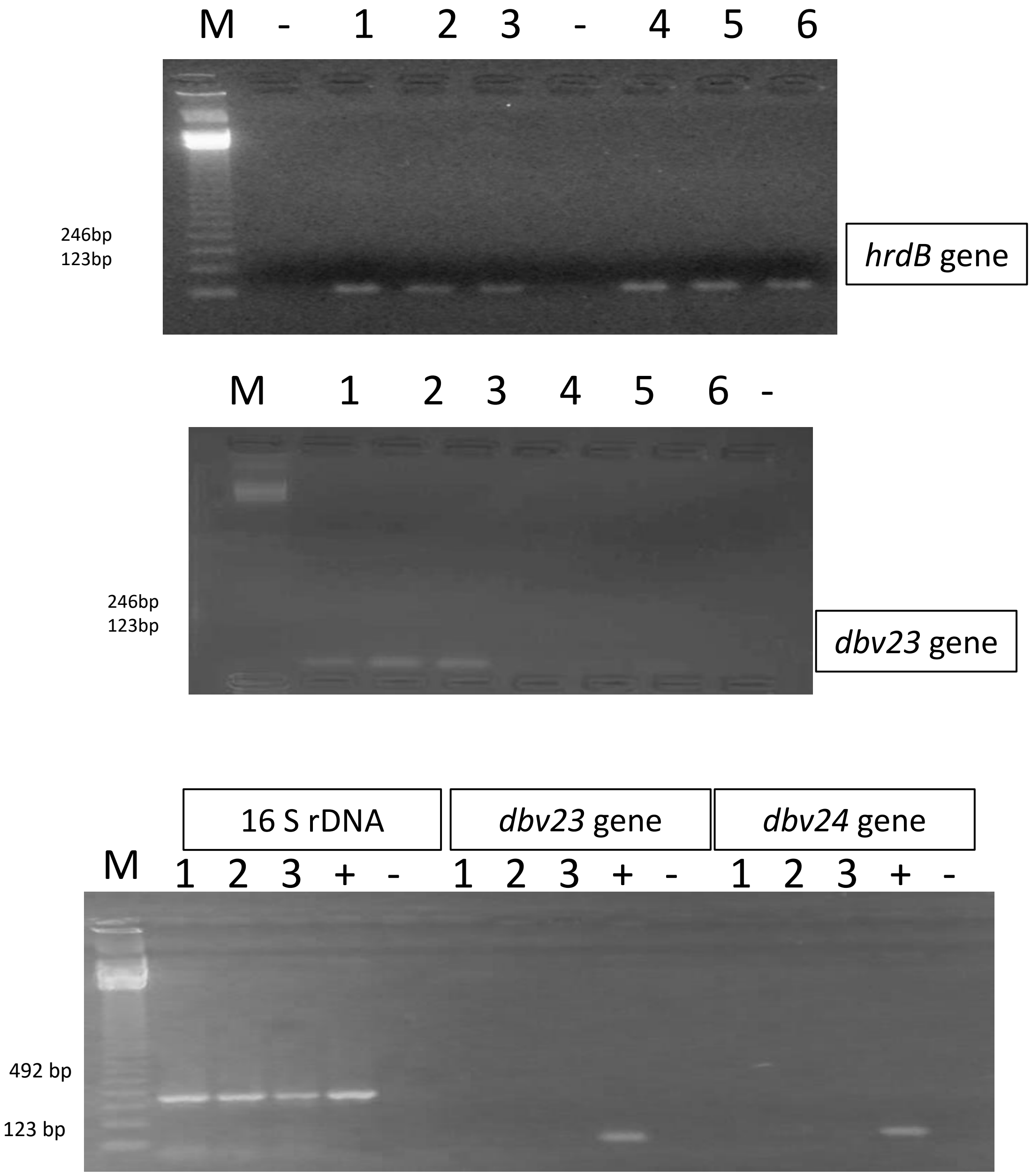

Supplementary Figure S3. PCR analysis of *hrdB*, 16S rDNA, *dbv23* and *dbv24* gene using cDNA prepared from WT (1) and Δdbv4 (4) strains without induction, WT (2) and Δdbv4 (5) strains induced with 0,5 µg/mL A40926 , WT (3) and Δdbv4 (6) strains induced with 0,5 µg/mL acA40926 M: 123bp DNA Ladder, Sigma-Aldrich. + and – indicate positive and negative controls.

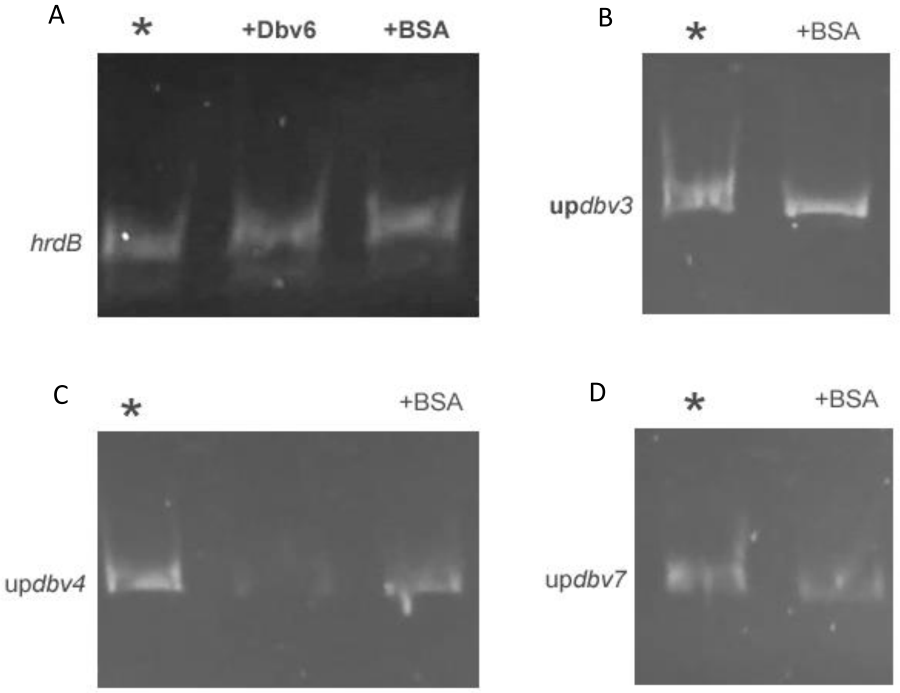

Supplementary Figure S4. Gel mobility shift assays of DNA regions.

In A the probe corresponds to the internal region of *hrdB*, that is not shifted by 15µg of Dbv6 and 15µg of BSA. In B, C and D the upstream region of *dbv*3, *dbv*4 and *dbv7* (D) were incubated in presence of 15µg of BSA. Lanes labeled with an asterisk contained the probe only. All lanes contained 50 ng of target DNA.

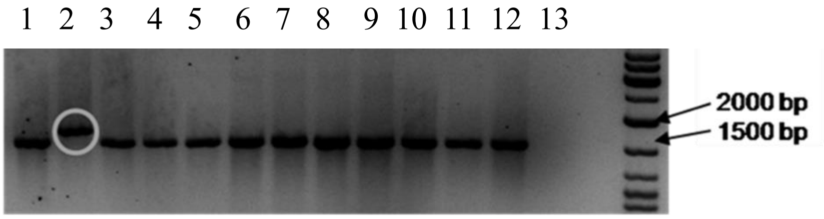

Supplementary Figure S5. PCR screening of 11 candidate double cross-over colonies (lanes 2-12) resulting from dbv22 construct. The PCR product of correct size (1.7 kb) is indicated by a circle. Lane 1 contains wild type genomic DNA used control template, while lane 13 contains the no-DNA negative control.
